# Knockdown of PDPN in astrocytes reduces hippocampal inflammation in T2DM mice

**DOI:** 10.3389/fimmu.2025.1503807

**Published:** 2025-01-22

**Authors:** Xiaohang Wang, Qianqian Wang, Zhensheng Cai, Chengming Ni, Huan Wang, Hui Liu, Yusong Zhao, Jinbang Wang, Subo Liu, Xueying Wang

**Affiliations:** ^1^ Department of Endocrinology, Affiliated Hospital of Yangzhou University, Yangzhou, China; ^2^ Department of Endocrinology, Zhongda Hospital, Institute of Diabetes, School of Medicine, Southeast University, Nanjing, China; ^3^ Institute of Translational Medicine, Jiangsu Key Laboratory of Integrated Traditional Chinese and Western Medicine for Prevention and Treatment of Senile Diseases, Medical College, Yangzhou University, Yangzhou, China; ^4^ Northern Jiangsu People’s Hospital, Northern Jiangsu People’s Hospital Affiliated to Yangzhou University, Yangzhou, China; ^5^ Department of Endocrinology, Shijiazhuang People’s Hospital, Shijiazhuang, China; ^6^ Department of Gland Surgery, Northern Jiangsu People’s Hospital, Northern Jiangsu People’s Hospital Affiliated to Yangzhou University, Yangzhou, China

**Keywords:** type 2 diabetes mellitus, vascular dementia, PDPN, astrocytes, inflammation

## Abstract

**Aims:**

Individuals with type 2 diabetes mellitus (T2DM) are at-risk for developing vascular dementia (VaD). Hyperglycemia leads to the activation of astrocytes. These activated cells produce proinflammatory mediators like cytokines or chemokines, that cause cerebrovascular damage. Previous sequencing showed *Pdpn*’s high expression in activated stellate cells and possible inflammation involvement. Our study aims to reveal its role in T2DM-induced hippocampal inflammation in VaD.

**Methods:**

Firstly, we will validate the expression of the *Pdpn* gene in T2DM astrocytes via qPCR and Western blot. Subsequently GFAP-specific promoter adeno-associated virus(AAV)carrying interfering sequence was used to knockdown the key gene in astrocytes of T2DM mice. Then the step-down test was conducted to assess the cognition level. The fluorescence intensities of IL-1β, IL-6, TNF-α, and TGF-β were measured via immunofluorescence assay to assess the level of inflammation in the brain after the key gene knockdown.

**Results:**

After the validation of transcriptome sequencing, the *Pdpn* gene was identified as a key gene upregulated in astrocytes from T2DM. Comparing to T2DM mice, knocking down *Pdpn* in astrocytes extended the latency and decreased the number of errors in T2DM mice, showing improved memory impairment. After the cognition assessment, the mice were euthanized, and the inflammatory factors associated to the VaD were detected by immunofluorescence. We showed that the fluorescence intensities of IL-1β, IL-6, TNF-α, and TGF-β1 in hippocampus were decreased after the *Pdpn* knocking down in astrocytes of T2DM mice.

**Conclusion:**

In summary, this study demonstrates that *Pdpn* exerts a novel player in T2DM-induced neuroinflammation and cognitive decline. Knocking down *Pdpn* in astrocytes shows a protective effect in hippocampal inflammation and VaD.

## Introduction

Type 2 diabetes mellitus (T2DM), a prevalent metabolic disorder characterized by chronic hyperglycemia, has emerged as a significant health burden worldwide. Beyond its direct impact on glucose metabolism, T2DM is increasingly recognized as a major risk factor for the development of vascular dementia (VaD), a form of cognitive decline primarily attributed to cerebrovascular dysfunction (1–3). The intricate interplay between T2DM and VaD underscores the urgent need to unravel the underlying molecular mechanisms that drive this pathological progression.

In response to metabolic stress, such as hyperglycemia associated with T2DM, astrocytes undergo a phenotypic transformation known as “reactive astrogliosis” (3). This process involves morphological, biochemical, and functional changes in astrocytes, which can have both beneficial and detrimental effects on neuronal function and survival. However, in the context of T2DM-induced VaD, reactive astrogliosis predominantly contributes to neuroinflammation and cerebrovascular damage (4, 5).

Activated astrocytes release a myriad of proinflammatory mediators, including cytokines and chemokines, which initiate and sustain an inflammatory cascade within the central nervous system (6–8). This inflammatory milieu disrupts the delicate balance between neuronal health and homeostasis, leading to a cascade of events that ultimately culminate in cognitive decline. Specifically, the proinflammatory environment fosters the development of atherosclerosis, blood-brain barrier disruption, cerebral small vessel disease, and other cerebrovascular abnormalities, all of which are hallmarks of VaD (9–12). Therefore, identifying the crucial molecule that augments the biological activity of astrocytes in diabetic conditions is paramount for mitigating hippocampal inflammation and delaying the progression of VaD.

In our previous study (13), we isolated another kind of stellate cells, islet stellate cells (ISCs), from fibrotic islets of Goto-Kakizaki (GK) Rats and Wistar Rats. These ISCs were consistently marked by Glial fibrillary acidic protein (GFAP), akin to astrocytes. To ascertain the transcriptome differences between Wistar Rat’s and GK Rat’s ISCs, we conducted RNA-seq, real-time PCR, and western blot validation on samples. Our findings revealed that Podoplanin (PDPN), encoded by the *Pdpn* gene, a mucin-type transmembrane sialoglycoprotein (13, 14), exhibited elevated expression in activated stellate cells. Further Gene Ontology (GO) biological process analysis unveiled that the *Pdpn* gene plays a crucial role in the significantly enriched term: “inflammatory response.” This suggests that *Pdpn* is implicated in the inflammatory processes associated with T2DM-induced brain injury. This suggests that PDPN, a glycoprotein known for its roles in cell migration, adhesion, and lymphatic vessel development, is also intricately involved in the inflammatory processes associated with Type 2 Diabetes Mellitus (T2DM)-induced brain injury. Research conducted by Fei (15) offers persuasive evidence to support this claim. Their study demonstrated that increased levels of PDPN were associated with elevated inflammatory responses, particularly in microglia, marked by heightened expression of interleukin-1β (IL-1β) and tumor necrosis factor-α (TNF-α).

In this study, our objective is to unravel the role of Pdpn in astrocyte activation induced by T2DM, with a particular emphasis on its contribution to hippocampal inflammation in the pathogenesis of VaD. By deciphering the molecular signatures underlying this pathological process, we aim to gain deeper insights into potential therapeutic targets that could alleviate the severe impact of T2DM on cognitive function.

## Methods

### Isolation and culture of astrocytes

To isolate and purify astrocytes, mice were euthanized and their brains were rapidly removed. The meninges were carefully stripped, and the brain tissue was minced and digested with trypsin. The resulting cell suspension was passed through a cell strainer to remove debris and then centrifuged to pellet the cells. For magnetic bead sorting, the cell pellet was resuspended in buffer containing anti-GFAP conjugated magnetic beads. Following incubation, the cells were passed through a magnetic column, where astrocytes bound to the beads were retained while other cell types were washed away. The purified astrocytes were then eluted from the column, counted, and plated onto poly-L-lysine coated flasks in Dulbecco’s modified Eagle’s medium/Ham’s F12 (DMEM/F12, Gibco) with 10% fetal bovine serum for further culture and experiments.

### Preparation of adeno-associated virus-sipdpn

To achieve targeted knockdown of PDPN expression in astrocytes *in vivo*, we utilized a GFAP-specific promoter-driven adeno-associated virus (AAV) vector. This vector was engineered to carry either a control interfering sequence (GFAP-AAV-con) or a *Pdpn*-interfering sequence (NM_010329, GFAP-AAV-pdpn^-^), obtained from Genechem Co., Ltd, Shanghai, China. GFAP serves as a biomarker for astrocytes, guiding the specificity of this approach.

The sense and anti-sense strands of siRNAs were: *Pdpn*, sense 5’-CCACGAUCACAAAGAACAUTT; antisense 5’-AUGUUCUUUGUGAUCGUGGTT.

### Animals

6-week-old male C57BL/6J mice were randomly allocated into 4 groups (n=8 per group). Following a 1-week acclimatization period, a low-dose STZ injection and a high-fat diet were administered to induce T2DM models, the following concise steps are employed: Feed the mice a high-fat diet for a specified duration (3 weeks) to induce insulin resistance and metabolic alterations characteristic of T2DM. After the high-fat diet period, administer a low dose of STZ (commonly in the range of 50 mg/kg body weight) via intraperitoneal injection. This dose is chosen to partially destroy the pancreatic beta cells, causing a moderate reduction in insulin secretion without causing full-blown diabetes as seen with higher doses. Following STZ injection, monitor blood glucose levels regularly to confirm the development of hyperglycemia, indicative of T2DM. Mice with persistently elevated blood glucose levels (typically >13.9 mmol/L or >250 mg/dL) are considered to have successfully developed the T2DM phenotype. Then, after 1 weeks, mice underwent intracranial stereotactic injection (hippocampus) with either GFAP-AAV-Pdpn^-^ virus or GFAP-AAV-con virus (1x10^11^ v.g per mouse), yielding the following groups: CON, T2DM, T2DM+AAV-con, and T2DM+AAV-pdpn^-^. All housing and experimental procedures adhered to the guidelines of the Yangzhou University Animal Care and Use Committee, ensuring compliance with institutional regulations and national animal welfare standards.

### Step-down passive avoidance test

The step-down apparatus consisted of a test box, an energized electric grid floor (10×10 cm), and an insulated jumping platform (YLS-3TB, Shandong Academy of Medical Sciences, Jinan, China), strategically placed in a corner. The three-day testing regimen began with a 5-minute acclimation period on day one, allowing mice to freely explore the uncharged box. On day two, the grid was activated at 36V and 0.5mA, and mice were gently positioned on the platform. Upon descending, mice encountered an electric shock, prompting immediate retreat to the insulated safety. On the final day, mice were retested, with their initial jump latency (step-down latency) and cumulative jumps (number of errors) within 3 minutes recorded for analysis.

### Quantitative real-time PCR

Total RNA was isolated using a rapid extraction method via SteadyPure Universal RNA Extraction Kit (AG21017, Accurate Biotechnology, China). For the cDNA synthesis, 1 mg of total RNA of each sample was reverse transcribed using HiScript II Q RT SuperMix for qPCR (R223- 01; Vazyme, China). Real-time PCR was performed on cDNA samples using the FastStart Universal SYBR Green Master (Roche) on Step One Plus system (Applied Biosystems, Foster City, CA, USA). Primers are described in **Supplementary Table S1**. The PCR settings used included denaturation (95°C for 2 min) and amplification steps repeated 40 times (95°C for 15s, 55°C for 30s, 72°C for 30s, and acquisition temperature for 15s). Analysis was conducted using the sequence detection software supplied with the instrument. For each sample, the delta delta Cycle of Threshold (ΔΔCT) (crossing point) values were calculated as the Ct of the target gene minus the Ct of the b-actin, assuming PCR efficiency equals to 1. Gene expression was derived according to the equation 2^–ΔΔCt^; changes in gene expression are expressed relative to levels of the other group.

### Western blotting

Briefly, flash-frozen tissues or cells were homogenized via radioimmunoprecipitation assay (RIPA) buffer containing protease inhibitors. BCA protein quantification was performed for tissue or cell lysates. PVDF membranes containing electrophoretically separated proteins from cells were probed with rabbit antibodies against Podoplanin n (EPR7073; Abcam), β-actin (81115-1-RR; Proteintech) treated with peroxidase (HRP)-conjugated goat anti-mouse or anti-rabbit IgG secondary antibody (BL001A; BL003A; Biosharp), and then visualized by enhanced Chemiluminescent horseradish peroxidase Substrate (Millipore).

### Immunofluorescence

Paraffin sections of 4 μm thickness were cut. Antigen repairing was performed in paraffin sections of brain tissues after dewaxing. Cell slides were fixed in 4% formaldehyde for 15min and permeabilized in 0.1% Triton X-100 for 5min. Then the tissue sections and cell slides were blocked by 10% goat serum (Servicebio, Wuhan, China) and incubated with primary antibodies at 4°C overnight. The primary antibodies used in this experiment were: rabbit anti-IL-6 (1:200; 12912; Cell signaling technology), rabbit anti-IL-1β (1:200; 16806-1-AP; Proteintech), mouse anti-TNF-α (1:200; 60291-1-Ig; Proteintech), rabbit anti-Transforming growth factor β-1 (TGF-β1; 1:200; 21898-1-AP; Proteintech). The secondary antibodies (SA00013-3, SA00013-4) for immunostaining were all purchased from Proteintech Co., Ltd. 4′,6′-diamidino-2-phenylindole (DAPI; Servicebio) was used for nuclei staining. Olympus FV1000 confocal microscopy system was used for image capture.

## Results

### The *Pdpn* gene is highly expressed in astrocytes of T2DM mice

**Figure 1:**
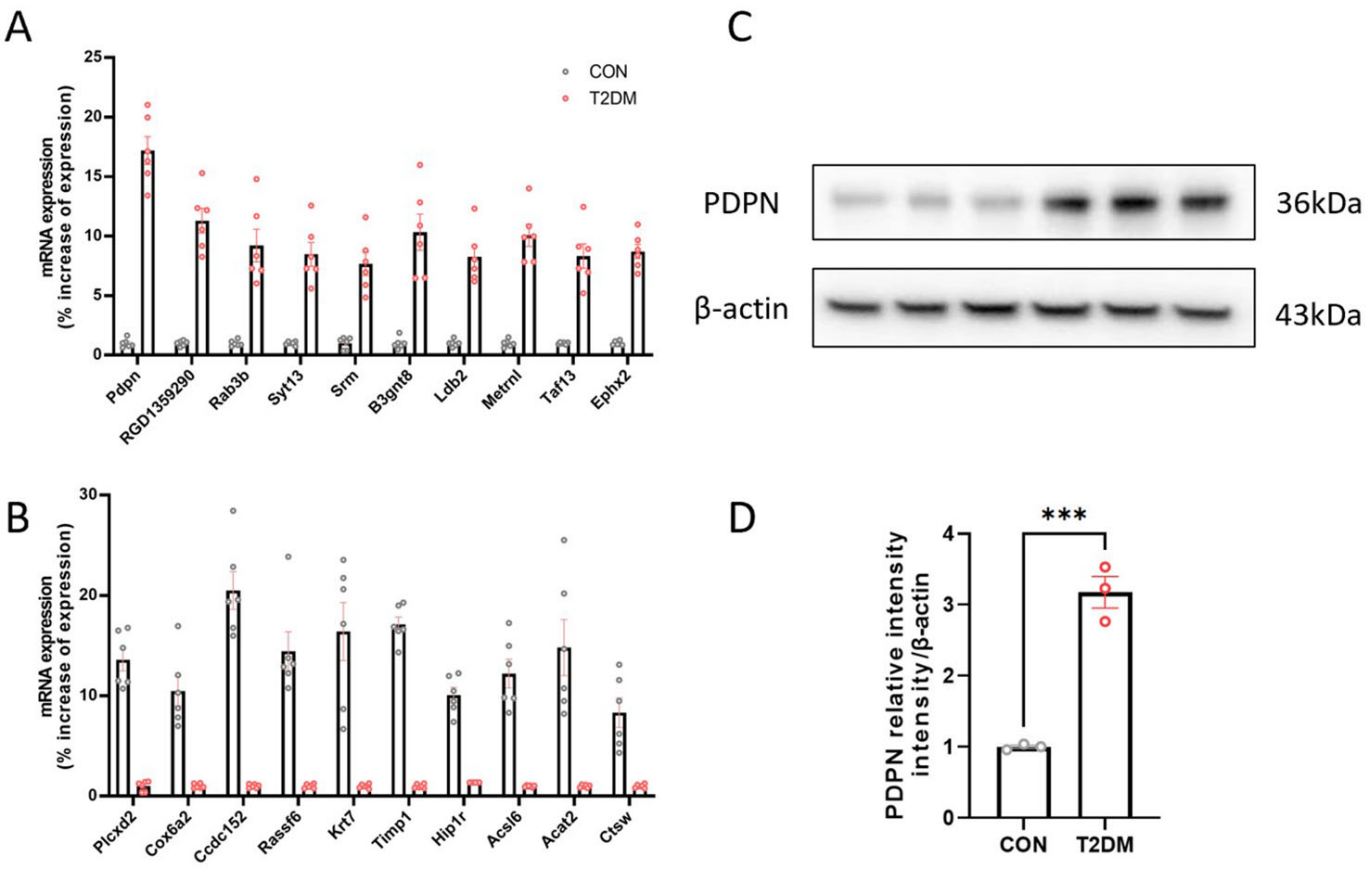
The Pdpn gene is highly expressed in astrocytes of T2DM mice. **(A)** Validation of 10 overexpressed differentially expressed genes in astrocytes of T2DM mice via real-time PCR. **(B)** Validation of 10 low-expressed differentially expressed genes in astrocytes of T2DM mice via real-time PCR. **(C, D)** Validation of high expression of PDPN in astrocytes of T2DM mice via western blot experiment. ***P < 0.001.

Based on previous transcriptome sequencing of activated and quiescent stellate cells (13), we identified 10 up-regulated genes and 10 down-regulated genes to explore the transcriptome alterations in activated astrocytes. Our results indicated that the *Pdpn* gene was the most notably overexpressed in the T2DM group. Subsequent qPCR (**Figures 1A, B**) and Western blot (**Figures 1C, D**) validations further confirmed that the *Pdpn* gene was significantly overexpressed in astrocytes of T2DM mice. Given the crucial role of *Pdpn* in inflammatory response, as revealed by GO biological process analysis, it suggests that *Pdpn* is implicated in the inflammatory processes that contribute to T2DM-induced brain injury.

### GFAP-specific knockdown of PDPN expression ameliorated memory deficits in T2DM mice in the step-down test

**Figure 2:**
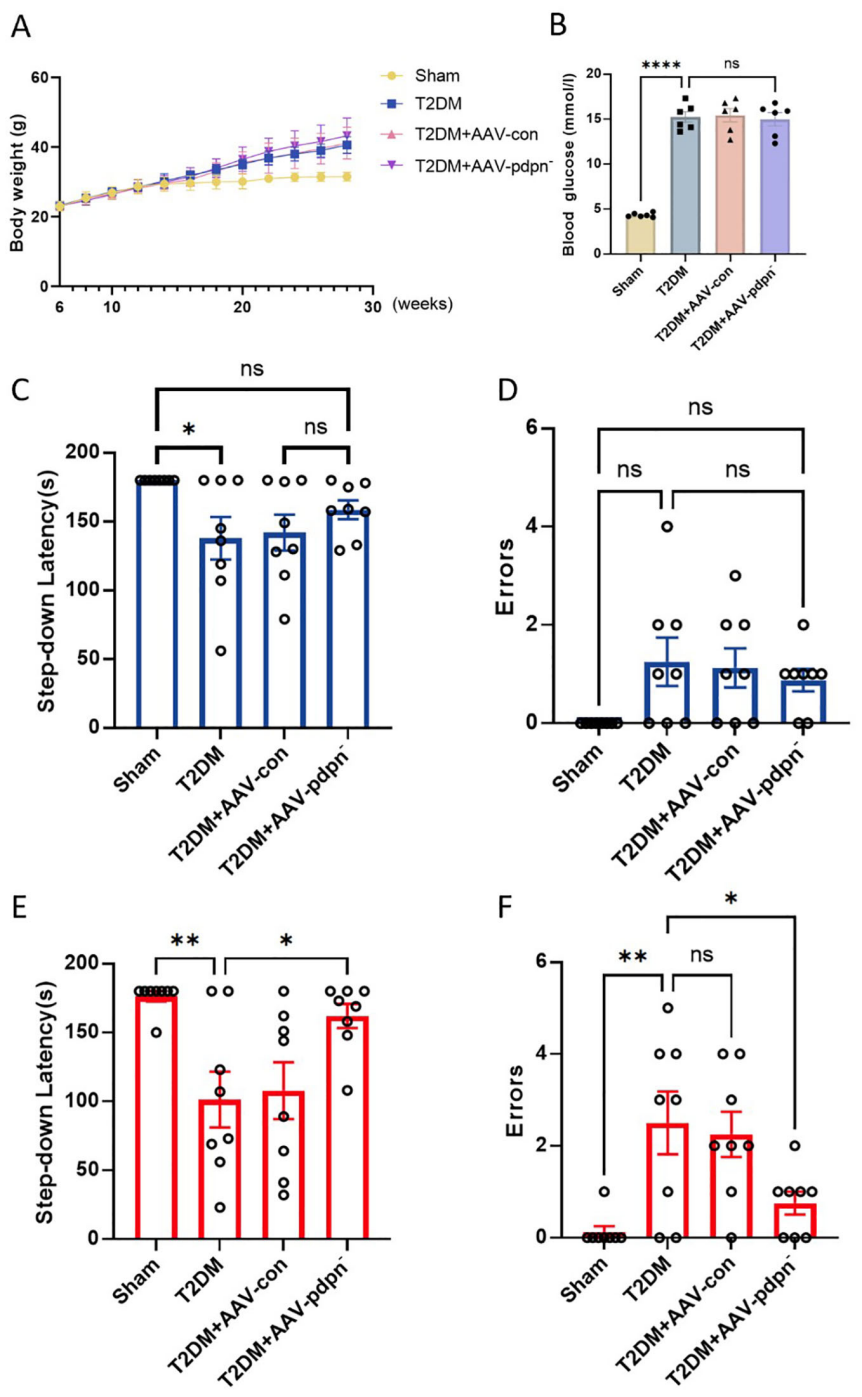
GFAP-specific knockdown of PDPN expression ameliorated memory deficits in T2DM mice in the step-down test. **(A)** The effect of GFAP-specific knockdown of PDPN expression in astrocytes on body weight, (n=8). **(B)** The results of FBG levels in each group of mice. **(C, D)** The comparison of step-down latency **(C)** and errors **(D)** among 12-week-old mice. **(E, F)** The comparison of step-down latency **(E)** and errors **(F)** among 28-week-old mice. Data are expressed as means ± SEM (n = 8), *P < 0.05, **P < 0.01, ****P < 0.0001. ns, no significance.

As shown in **Figure 2A**, all four groups of mice maintained consistent body weights before the 8th week. However, the group with a high-fat diet and induced with low-dose STZ for T2DM experienced a significant increase in body weight after the 8th week (**Figure 2A**). Furthermore, these T2DM groups exhibited significantly elevated fasting blood glucose levels compared to the CON group, with statistically significant differences as depicted in **Figure 2B**.

In comparison to the 12-week-old T2DM group, the T2DM+AAV-pdpn^-^ group demonstrated an extended latency period. Additionally, this group exhibited a reduction in errors during the step-down passive avoidance test. Conversely, no statistically significant differences were discernible between the T2DM group and the T2DM+AAV-con group (**Figures 2C, D**).

Analogous findings were replicated in 28-week-old mice, where the T2DM+AAV-pdpn^-^ group displayed a markedly longer latency period in comparison to the T2DM group (**Figure 2E**). Furthermore, the T2DM+AAV-pdpn^-^ group had significantly fewer errors (**Figure 2F**).

These findings revealed that T2DM mice displayed compromised performance in the step-down test as early as 12 weeks of age, progressing to severe memory deficits by 28 weeks. Notably, the knockdown of PDPN expression significantly ameliorated the memory impairment observed in 28-week-old T2DM mice.

### GFAP-specific knockdown of PDPN expression alleviates inflammation in the hippocampus of T2DM mice

**Figure 3:**
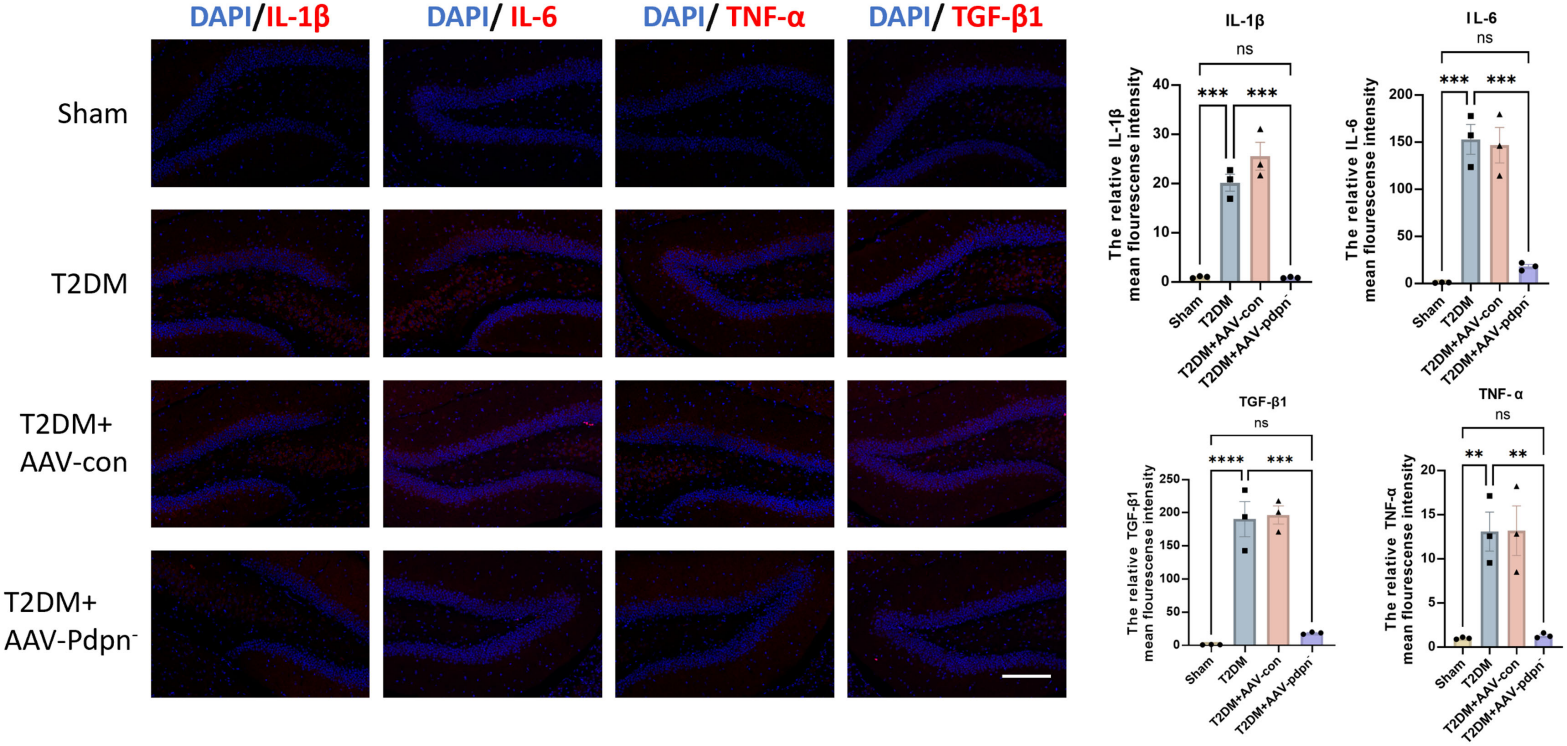
GFAP-specific knockdown of PDPN expression alleviates inflammation in the hippocampus of T2DM mice. Representative images and statistical analysis graphs of IL-6, IL-1β, TNF-α, and TGF-β1 expression in hippocampus of T2DM mice via immunofluorescence. Magnification 400 ×; Scale bar, 100 μm; **P < 0.01, ***P < 0.001, ****P < 0.0001. ns, no significance.

Given the established association between the *Pdpn* gene and the biological process of inflammatory response, we hypothesized that knockdown of *Pdpn* in astrocytes would reduce inflammation levels in the hippocampus of T2DM mice, thereby ameliorating their memory impairment. To validate this hypothesis, we performed immunofluorescence staining for IL-1β, IL-6, TNF-α, and TGF-β1, inflammatory markers closely linked to endothelial cell injury, on hippocampal sections from four groups of mice. Our results demonstrated that knockdown of *Pdpn* significantly decreased the expression of these four inflammatory indicators, suggesting a reduction in inflammation within the hippocampus (**Figure 3**). This finding supports our initial premise that modulating *Pdpn* expression in astrocytes can mitigate the inflammatory milieu contributing to memory deficits in T2DM mice.

## Discussion

The present study sheds light on a novel and intriguing role of *Pdpn* in the context of T2DM-associated neuroinflammation and cognitive decline, particularly focusing on its upregulation in astrocytes within the hippocampus. Our results revealed a significant increase in PDPN expression in astrocytes isolated from T2DM mouse models, suggesting a potential link between this glycoprotein and the pathological processes underlying diabetic encephalopathy.

The hippocampus, a crucial brain region for learning, memory, and cognitive function, is particularly vulnerable to the deleterious effects of T2DM (16, 17). Chronic hyperglycemia and associated metabolic derangements in T2DM lead to a cascade of inflammatory responses, characterized by the activation of microglia and astrocytes, which contribute to neuroinflammation and neuronal damage. The presence of inflammatory factors, fueled by obesity or diabetes, holds a direct correlation with the pathogenesis of Alzheimer’s disease (AD). This is underscored by the heightened levels of IL-6, IL-1β, and TNF-α observed in both the plasma and the central nervous system of patients diagnosed with AD (18). Our findings of elevated *Pdpn* levels in astrocytes within the hippocampus of T2DM mice align with this inflammatory milieu and suggest that *Pdpn* may be a key player in mediating these inflammatory processes.

Podoplanin, is highly expressed in T2DM stellate cells detected from the RNA-seq (13). The observed correlation between increased PDPN expression and hippocampal inflammation underscores the need to further investigate the functional significance of this upregulation. To this end, we employed a gene knockdown approach to specifically target PDPN in T2DM mice. Remarkably, the downregulation of PDPN led to a significant improvement in cognitive function, as evidenced by enhanced performance in cognitive tasks. This finding underscores the detrimental role of PDPN in T2DM-induced cognitive decline and highlights its potential as a therapeutic target.

Furthermore, the amelioration of hippocampal inflammation following PDPN knockdown provides mechanistic insights into the beneficial effects observed. By reducing PDPN levels, we may have disrupted inflammatory signaling pathways that are activated in response to T2DM-induced stress, thereby mitigating the neuroinflammatory response and protecting neurons from damage. This is particularly relevant given the growing body of evidence linking neuroinflammation to cognitive impairment in T2DM.

The precise molecular mechanisms underlying the PDPN-mediated inflammatory response in T2DM astrocytes remain to be fully elucidated. However, several possibilities can be speculated upon. PDPN, as a transmembrane glycoprotein, has been shown to interact with various signaling molecules and receptors, including CLEC-2 and ezrin/radixin/moesin (ERM) proteins, which are involved in cell adhesion, migration, and signaling (19–21). In the context of T2DM, these interactions may be dysregulated, leading to aberrant activation of inflammatory pathways. Additionally, PDPN has been implicated in the regulation of immune cell function, including the recruitment and activation of macrophages and lymphocytes (22, 23). Numerous studies have confirmed the association between PDPN-positive cells and inflammatory factors secreted by various immune-related cells (24, 25). In the brain, astrocytes can adopt an immune-like phenotype in response to injury or stress, and PDPN may play a role in modulating this response in T2DM.

The present study, while offering novel insights into the potential roles of PDPN in GFAP-positive cells, is not devoid of limitations. Firstly, although GFAP-positive cells are predominantly astrocytes within the nervous system, their presence in other organs such as the liver and pancreas suggest a broader distribution and potential functional relevance. Via the utilization of stereotaxic injection technique, the findings and conclusions are specifically pertinent to the hippocampal region in this study. Future studies are thus warranted to delve deeper into the metabolic consequences of PDPN alteration in these non-neural GFAP-positive cells. Secondly, a notable limitation of this research lies in the lack of a comprehensive investigation into the underlying mechanisms by which PDPN influences astrocyte inflammation.

In conclusion, our study identifies PDPN as a novel player in T2DM-induced neuroinflammation and cognitive decline. The upregulation of PDPN in astrocytes within the hippocampus of T2DM mice and its correlation with hippocampal inflammation suggest a functional role in mediating these pathological processes. Importantly, the downregulation of PDPN through gene knockdown improves cognitive function and reduces hippocampal inflammation, highlighting its potential as a therapeutic target. Future studies should aim to elucidate the precise molecular mechanisms underlying the PDPN-mediated inflammatory response in T2DM astrocytes and explore the therapeutic potential of targeting PDPN in the treatment of diabetic encephalopathy.

## Data Availability

The datasets presented in this study can be found in online repositories. The names of the repository/repositories and accession number(s) can be found below: platform:baiduyun https://pan.baidu.com/s/1vyZaaV5jjHN-9ALRiq6ovw?pwd=te95 key:te95.
